# The State of the Science on Chronic Comorbidities and Aging in Children and Adolescents with perinatally-acquired HIV

**DOI:** 10.1007/s11904-025-00761-0

**Published:** 2025-12-06

**Authors:** Modupe O. Coker, Ryan Kreutzberg, Nadia A. Sam-Agudu, Eilleen Macodiyo, Jibreel Jumare, Juliette Madan, Vaishali Singhal, Zhigang Li, Reuben Robbins, Stephanie Shiau

**Affiliations:** 1 Department of Basic and Translational Sciences, Penn Dental Medicine, University of Pennsylvania, Philadelphia, PA USA; 2 Department of Biostatistics and Epidemiology, Rutgers School of Public Health, Rutgers University, Piscataway, NJ USA; 3 Department of Epidemiology, Geisel School of Medicine, Dartmouth, NH USA; 4 International Research Center of Excellence, Institute of Human Virology Nigeria, Abuja, Nigeria; 5 Global Pediatrics Program and Division of Infectious Diseases, Department of Pediatrics, University of Minnesota Medical School, Minneapolis, MN USA; 6 Department of Paediatrics and Child Health, University of Cape Coast School of Medical Sciences, Cape Coast, Ghana; 7 Rutgers State University of New Jersey, Biomedical Engineering Graduate Program, School of Graduate Studies, Newark, United States; 8 University of Maryland, Baltimore, MD USA; 9 Department of Psychiatry, Dartmouth Hitchcock Medical Center, Lebanon, NH USA; 10 Department of Interdisciplinary Studies, Rutgers School of Health Professions, Newark, NJ USA; 11 Department of Department of Pediatric Dentistry and Community Health, Rutgers School of Dental Medicine, Newark, NJ USA; 12 Department of Biostatistics, University of Florida, Gainesville, FL USA; 13 Department of Psychiatry, Columbia University, New York, NY USA

## Abstract

The availability and global scale-up of suppressive antiretroviral therapy (ART) has transformed HIV from a fatal infection to a chronic disease. While long-term survival is a positive development for people living with HIV (PLWH), the increased life expectancy comes with age-related comorbidities. These comorbidities affect PLWH at relatively earlier ages than the general population, and examples include cardiometabolic conditions, renal toxicity, lung and respiratory dysfunction, neurocognitive deficits, malignancy, and oral/dental pathology. Anticipatory management of early markers of non-communicable diseases (NCDs) can be especially advantageous for children and adolescents with perinatally-acquired HIV (CAPHIV), the vast majority of whom live in low-and middle-income countries. However, evidence for the mechanisms underlying age-related comorbidities in PLWH and implications for CAPHIV represent a still-emerging area of investigation. In this article, we review the current literature on comorbidities and age-related conditions experienced by CAPHIV, discuss the role of inflammation and chronic immune activation, and highlight accelerated biological aging and/or disruptions to the microbiome as underlying mechanisms. We recommend that HIV clinical care and health policy should reflect evidence on aging and comorbidities to optimize growth, development, and long-term health for CAPHIV globally.

**Supplementary Information:**

The online version contains supplementary material available at 10.1007/s11904-025-00761-0.

## Introduction

With increasing access to antiretroviral therapy (ART) for women and infants, programs to prevent perinatal transmission of HIV have expanded and become more accessible, globally. This has led to a reduction in the number of infants with perinatally-acquired HIV (PHIV) and a growing number of children (0–9 years) living with PHIV who are reaching adolescence (10–19 years of age) and adulthood [1]. While access to effective ART has transformed HIV from a fatal infection to a chronic disease, chronicity comes with its own health issues. In adults living with HIV, risk of age-related comorbidities has increased with increasing life expectancy [2]. Further, due to the combination of HIV, ART, environmental factors, and aging processes, people living with HIV (PLWH) are at higher risk for age-related chronic conditions compared to their HIV-seronegative counterparts [3]. These chronic comorbidities include cardiovascular disease, metabolic syndrome, renal and liver disease, osteoporosis and fractures, neurocognitive disorders, and metabolic syndrome as well as geriatric syndromes and frailty [4].

Age-related chronic conditions can also be seen in children and adolescents with PHIV (CAPHIV), who are exposed to HIV from *in utero* and birth [5–7]. Studies suggest unique differences in HIV, ART exposure, and ART treatment during periods of critical development can exacerbate co-morbidities in this younger population compared to people acquiring HIV in adulthood [8, 9]. Given increasing numbers of CAPHIV, there is a growing need to understand the impact that HIV will have on their future health and development as they age into adulthood.

Understanding the mechanisms underlying chronic comorbidities in CAPHIV is particularly crucial in low- and middle-income countries (LMICs), where the vast majority of CAPHIV reside [10]. In these settings, perinatal HIV transmission remains a major driver of new infections, and healthcare infrastructure often faces limitations in providing long-term disease management [11]. Given the high burden of pediatric HIV in sub-Saharan Africa (SSA) and other LMICs, identifying the biological and immunological pathways contributing to comorbidities is essential to develop targeted screening and intervention programs. Mechanistic studies in these populations can help clarify how chronic inflammation, immune activation, and other factors accelerate aging-related conditions, guiding efforts to improve long-term health outcomes and optimize treatment strategies.

The purpose of this narrative review is to describe the current state of the science on chronic comorbidities experienced by CAPHIV, and highlight disruptions to the microbiome and accelerated biological aging as contributing mechanisms underlying the development of these comorbidities.

## Methodology

To synthesize current evidence on comorbidities and health outcomes among CAPHIV, a systematic literature search was conducted using the PubMed/MEDLINE database. The search spanned January 1, 2015, to December 31, 2024, to capture recent trends and contemporary findings relevant to this population.

Studies were selected using the following structured search criteria:

- Articles mentioning HIV in the title or abstract and including infants, children, adolescents, or youth, with a specific focus on perinatally or vertically-acquired infection.
- Studies addressing comorbidities such as cardiometabolic disease, diabetes, metabolic dysfunction, growth and development, cognitive and mental health, bone and skeletal health, oral and dental health (including HPV, EBV, CMV), respiratory and lung disease, hepatic and renal outcomes, cancer, and antiretroviral-related effects.
- Filters applied: Only studies involving humans, published in English, including participants aged 0–18 years and providing free full text were considered. Preprints, protocols, review articles, and case series were excluded.
- To ensure relevance and rigor, only studies with comparative elements were included.

The following criteria were used to select studies:

(HIV[Title/Abstract]) AND ((infants) OR (children) OR (adolescents) OR (youth)) AND ((perinatally-infected) OR (perinatally-acquired) OR (perinatally acquired) OR (perinatally infected) OR (vertically infected)) AND ((comorbidities) OR (cardiometabolic) OR (diabetes) OR (glucose) OR (metabolic) OR (aging) OR (cardiac) OR (oral) OR (HPV) OR (EBV) OR (CMV) OR (dental) OR (development) OR (growth) OR (anthropometric) OR (renal) OR (hepatic) OR (antiretroviral) OR (cancers) OR (cognitive) OR (brain) OR (mental) OR (bone) OR (skeletal) OR (lung) OR (respiratory)) AND ((excludepreprints[Filter]) AND (fft[Filter]) AND (humans[Filter]) AND (female[Filter] OR male[Filter]) AND (2015/1/1:2024/12/31[pdat]) AND (english[Filter]) AND (allchild[Filter])) NOT (Profile[Title]) NOT (protocol[Title]) NOT (This review[Title/Abstract]) NOT (review) NOT (Men[Title]) NOT (case series[Title/Abstract]) AND ((comparative study[Title]) OR (compared to[Text Word]) OR (relative to[Text Word]))

Of the 366 studies resulting from the search, 40 studies met the following criteria (i) original studies excluding reviews involving clinical outcomes and comorbidities; and (ii) involving youth (children and adolescents) who perinatally-acquired HIV compared to uninfected counterparts, excluding several studies comparing only children HIV-exposed-uninfected with uninfected. For several studies however, it was not clear whether the children were living with perinatally-acquired HIV. Twenty additional studies were identified upon further evaluation of reviews and cross-references for specific clinical outcomes. A summary table of included original studies, detailing year of study/publication, country, age group, methods, and findings, is provided in Supplementary Table 1.

## Comorbidities Related To Aging in Children and Adolescents with Perinatally-Acquired HIV

**Fig. 1:**
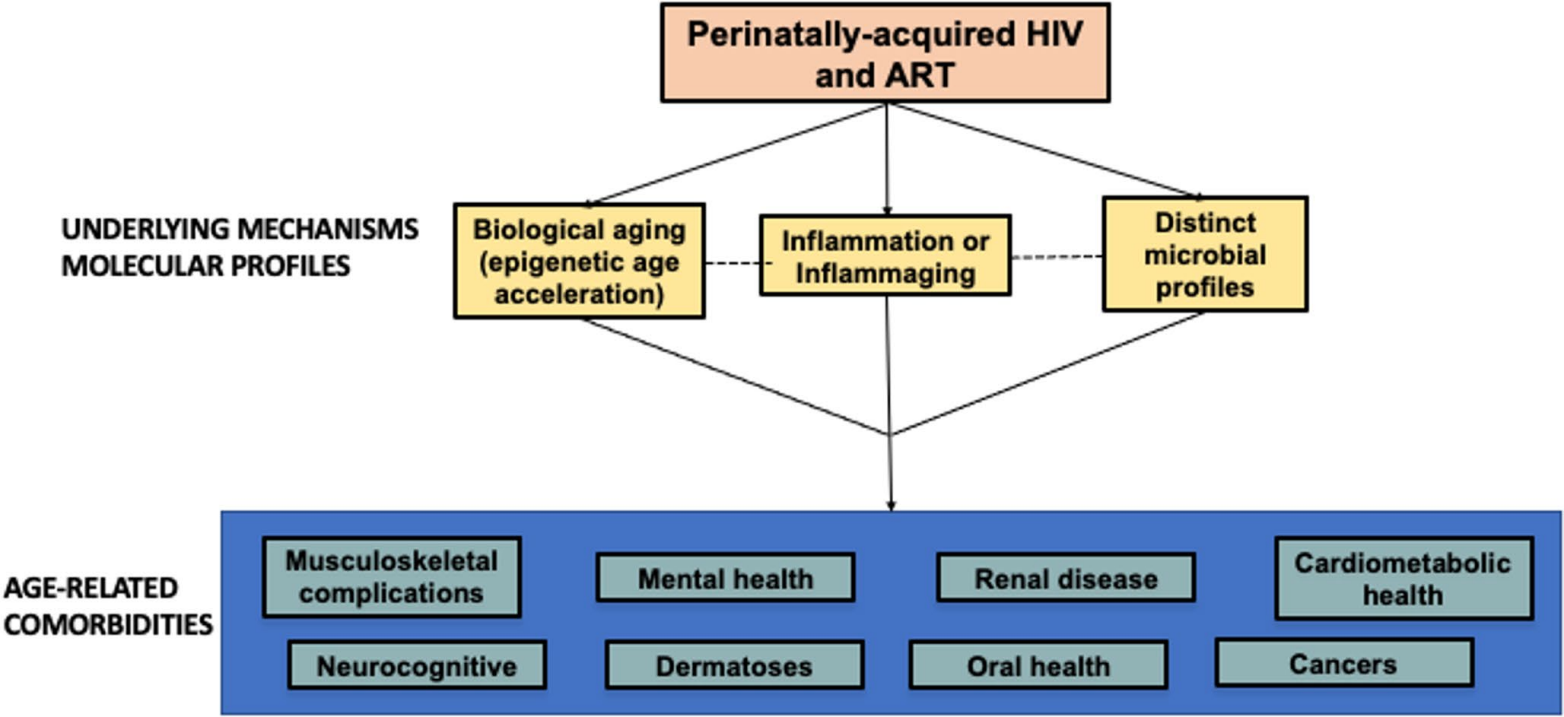
Aging with Perinatally-acquired HIV Infection. Molecular profiling via epigenetic aging, the microbiome, immune activation, and inflammation is crucial for elucidating the mechanisms driving chronic conditions in children and adolescents living with HIV. Future research exploring these intricate relationships may uncover new therapeutic targets and interventions aimed at mitigating the burden of chronic comorbidities in individuals with HIV

HIV-related comorbidities in children and adolescents can present in varied forms, each with their own challenges. The following sections provide an update on the following comorbidities among CAPHIV: cardiometabolic conditions, renal toxicity, lung and respiratory dysfunction, growth and developmental disorders, musculoskeletal disorders, mental health disorders, neurocognitive disorders, cancer/malignancies, and other diseases such as dermatologic and oral/dental pathologies (Fig. 1).

### Cardiometabolic Conditions

Multiple studies have shown an increased prevalence of cardiac dysfunction, characterized by structural (left ventricular dysfunction, increased carotid intima-media thickness, changes in chamber dimensions) and functional (reduced ejection fraction) cardiac abnormalities in CAPHIV across different ages and in the US and SSA [12–14]. These abnormalities are thought to arise from a combination of chronic immune activation, ongoing inflammation, and potential toxicities from specific ART regimens [15]. Additionally, CAPHIV often exhibit elevated carotid intima thickness and increased arterial stiffness, both indicative of early vascular aging and heightened cardiovascular risk [16, 17]. This endothelial dysfunction and arterial remodeling could be a result of reduction in nitric oxide bioavailability with chronic HIV immune activation or ART-associated metabolic derangements that can exacerbate lipid imbalances, amplifying the cardiovascular risk profile [18, 19].

Insulin resistance represents another significant cardiometabolic issue observed in CAPHIV, which is linked to both HIV infection and metabolic side effects of ART. Insulin resistance not only predisposes individuals to Type 2 diabetes but also contributes to endothelial dysfunction, inflammation, and dyslipidemia, fostering a milieu conducive to cardiovascular disease [20]. Since early cardiometabolic markers are observed in CAPHIV, it remains important to intervene early and provide comprehensive care for this population throughout the lifespan [21].

### Renal Toxicity

Renal toxicity remains a critical issue in CAPHIV, even in the era of ART. Before the widespread use of ART, HIV-associated nephropathy was the most common form of chronic kidney disease in this population, particularly among CAPHIV in Africa [22]. However, with the advent of ART, the incidence of HIV-related nephropathy has significantly decreased, altering the prognosis from progression to end-stage kidney disease to one that is compatible with long-term survival [22]​​. Despite this progress, studies have documented that renal abnormalities, including microalbuminuria and proteinuria, still persist in a substantial proportion of CAPHIV, with one study reporting these conditions in 20.4% of CAPHIV ages 7–16 years [23]​​. Risk factors contributing to renal dysfunction in CAPHIV include older age, lower CD4 + T-cell percentage, and higher HIV-1 viral load, with studies suggesting a particular vulnerability in sub-Saharan Africa, where the burden of HIV is heaviest [24]​​.

Tenofovir disoproxil fumarate (TDF), a widely used component of antiretroviral therapy (ART) regimens, has been associated with renal toxicity in CAPHIV. Adult studies have shown a relationship between TDF exposure and renal dysfunction but fewer were conducted in CAPHIV. For example, a prospective cohort study in adults identified an independent association between the duration of TDF use and the development of proteinuria, where longer exposure correlated with a higher risk [25]. Similarly, in children and young adults who acquired HIV in early life, the use of TDF has been linked to an increase in subclinical markers of renal impairment, including anion gap and decreased estimated glomerular filtration rate (eGFR) [26, 27]. These findings underscore the importance of monitoring renal function in CAPHIV treated with TDF and taking a prudent approach in the selection of ART regimens to minimize potential renal adverse effects. It is important to note that tenofovir alafenamide (TAF), a newer formulation, has demonstrated a significantly lower risk of renal toxicity in children and adolescents [28, 29].

### Lung and Respiratory Dysfunction

Lower nadir CD4 counts have been associated with an increased risk of chronic lung impairment, suggesting that historical immunosuppression may contribute to long-term respiratory complications in this population [30]. With the widespread use of ART, the burden of lung and respiratory dysfunction in CAPHIV remains an enduring challenge. Although ART initiation has reduced the incidence of acute respiratory infections, chronic lung diseases, such as small airway disease, continue to persist [31, 32]. A review suggested that lungs could act as a reservoir for HIV, contributing to persistent pulmonary immune dysregulation despite ART [33]. This hypothesis was supported by another study providing evidence that even with early ART initiation, a subset of children still displayed abnormal spirometry, indicative of lung dysfunction [31].

Irrespective of ART, factors that aggravate lung and respiratory conditions in CAPHIV include early-life pneumonia, immune suppression, and socio-environmental exposures such as air pollution [34]. Risk factors extend beyond the virus itself, encompassing environmental and socio-demographic elements. Some studies show that a history of pulmonary infections, undernutrition, and air pollution exposure are pivotal in determining lung health outcomes in CAPHIV [8, 35]. These factors, along with the immunological challenges posed by HIV, set the stage for a spectrum of respiratory conditions, including obstructive and restrictive lung diseases [31, 35–37].

The geographic epicenter of many chronic lung complications is in LMICs, particularly sub-Saharan African countries [32, 37]. These regions have high rates of early-life pneumonia, malnutrition, and exposure to indoor air pollution, which are compounded by delayed initiation of ART, further increasing the burden of respiratory diseases in CAPHIV [38]. The evidence points to a need for comprehensive care strategies, ongoing surveillance, and specific interventions to support lung health in CAPHIV, particularly in resource-limited settings [8, 31, 32].

### Growth and Developmental Disorders

Impaired growth and developmental disorders have been reported in CAPHIV [39]. Infancy, childhood, and adolescence are critical time periods in which preventive measures and interventions are needed to avoid long-term impairment in growth and development. Studies have shown that the greatest growth discrepancy for CAPHIV is in the first two years of life, even when adjusting for confounding factors [40–42]. In comparison to children and adolescents without HIV, CAPHIV have lower weight, height, and head circumference measurements throughout childhood and have been found to have abnormal body composition, characterized by a lower proportion of lean body mass, reduced muscle mass, and an increased proportion of fat [43]. Across several regions and age groups, studies have reported approximately 20% of CAPHIV have anthropometric measures below the 5th percentile [44–46].

HIV-associated growth failure is caused by multiple factors, including poor nutrition, malabsorption, increased metabolism, and growth inhibition mediated by cytokines [44, 47]. Viral proteins could damage the hypothalamus and pituitary glands, disrupting the hormonal regulation of growth and development. During the acute phase of malnutrition or malabsorption, levels of IgG can decrease substantially, which is a significant predictor of mortality in CAPHIV [48, 49]. In addition, there is preliminary evidence that the metabolic demands of persistent and recurrent infections and the effects of immune activation and inflammation on the production, transmission and action of insulin-like growth factor-1 (IGF-1), a hormone that regulates growth hormone activity, can lead to growth failure in CAPHIV [50].

Recovery in growth does not seem to occur when nutrition improves, suggesting that perinatal HIV exposure and infection can have long-term effects on the developing physiology [41]. While many of these growth disorders are likely to be reversed by undergoing ART and with adequate access to nutrition, especially prior to the critical period of growth and development during adolescence [51–53], reports of compromised growth outcomes still exists [54–56]. Additional knowledge of critical growth factor pathways that are affected by the direct and indirect effects of HIV infection on target bones and tissues may lead to an improved understanding of growth failure pathogenesis in CAPHIV.

### Musculoskeletal Diseases

There have been reports of bone loss in PLWH that affects alveolar, spine and long bones [26, 57–62]. As previously discussed, in CAPHIV, increasing data suggests that HIV is associated with impaired or compromised growth [55]. Similarly, increased risk of bone loss and fractures is observed in adults living with HIV on ART [63]. Youth in particular are in a critical developmental period for musculoskeletal development and bone density, as they have the best opportunity to “invest” in their bone health by achieving an optimal peak bone mass [64–67]. There have been multiple reports of low BMD among CAPHIV. The increased risk of bone loss and fracture is of concern because impaired growth has a rippling effect on overall systemic developments and the risk of fracture in adulthood is multiplied for CAPHIV on lifelong ART. Whether this bone loss is associated with HIV or ART is unclear. Even following ART initiation, treatment has been implicated in exacerbating preexisting bone damage [68]. There is some evidence to suggest that ART-driven immune recovery in immunocompromised PLWH can cause additional bone loss within 6-12 months of ART initiation [59].

### Mental Health Disorders

Compared to their counterparts without HIV, findings show that there is a higher prevalence of psychiatric disorders in CAPHIV [69–73]. A high prevalence of mental health challenges, including depression, anxiety, attention-deficit/hyperactivity disorder (ADHD), and behavioral disorders, have been reported among CAPHIV [70, 72, 74]. The high prevalence of mental health disorders among CAPHIV is thought to be multifaceted, with various factors such as sex, CD4 + count, living status of parents, caregiver status, cognitive function, stressful life events, and social support (or lack thereof) - all implicated [70, 71, 73]. Perceived HIV stigma can also affect mental health; as stigma increases considerably with age, mental health declines among CAPHIV [75]. Beyond these social and behavioral factors, there are clear gaps in understanding the underlying distinct biological and immunological mechanisms, particularly along the gut microbiome-brain axis [76, 77], that are needed to prevent mental health sequalae and support appropriate interventions.

### Neurocognitive Deficits

While some studies have reported minimal differences in cognitive outcomes among CAPHIV compared to healthy peers [71], CAPHIV have been shown by several others [5, 6, 78, 79], to experience neurocognitive challenges in higher proportions than those without HIV. Research has shown that adolescents with HIV, even those who began ART at an early age (in the first year of life), can experience neurocognitive deficits, affecting aspects such as working memory, executive function, and processing speed [80–82]. Of note, a recent cross-sectional study in Netherlands, found that when compared to siblings uninfected with HIV, CAPHIV aged 5–18 exhibited compromised functioning in various neurocognitive-related areas, including attention deficits, sensory processing, social-emotional well-being, and diminished health-related quality of life, as reported by caregivers and teachers [78]. While several neurocognitive screening tools such as BRACE [83], Neuroscreen [84], NIH Toolbox [85] have been validated for use in various regions burdened by populations with HIV, there is an important need to develop and validate culturally sensitive, region-specific tools that can more accurately assess neurocognitive impairment in all settings, including in sub-Saharan Africa, particularly for CAPHIV. Additionally, ongoing efforts are required to ensure these tools are accessible and feasible for use in resource-limited settings, and that they can effectively track neurocognitive changes over time to inform clinical management and therapeutic interventions.

Potential mechanisms for neurocognitive deficits include accelerated aging processes including chronic neuroinflammation, oxidative stress, impaired autophagy and ultimately neurodegeneration. More studies are necessary to better understand the relationship between accelerated aging and cognitive deficits, particularly in CAPHIV.

### Cancers/Malignancies

CAPHIV remain susceptible to non-AIDS-defining cancers (such head and neck cancers, Hodgkins lymphoma, liver cancers), most due to viral co-infections, including oncogenic viruses. Understanding this burden is particularly important because youth with pediatric cancers have limited access to oncological services and experience delayed access to treatment [86].

#### Human Papilloma Virus (HPV)-associated Lesions and Cancers

The prevalence of human papilloma virus (HPV)-associated lesions and cancers in PLWH remains elevated [87–89] and includes mostly cervical, vaginal, anal and penile cancers, with oral/oropharyngeal cancers being rarer in comparison (https://pubmed.ncbi.nlm.nih.gov/40086453/). Notably, ART has not consistently been shown to protect against any of these invasive carcinomas. Even in the ART era, PLWH have increased risk of oral, cervical, anal, and penile HPV infection and persistence of high-risk-HPV subtypes. Most of the data on this association comes from high-income countries [88, 90], and largely from populations of young adults or older individuals. As children living with HIV reach adolescence and become sexually active, they are at risk of acquiring HPV infection; reports from adolescent females with PHIV have been shown to have a higher prevalence of high-risk HPV and abnormal cervical cytology compared to adolescents without HIV/PHIV, after adjusting for age, sexual history and pregnancy [91]. While there are limited studies of HPV incidence and clearance in youth [92, 93], evidence from adult cohorts suggests that HIV infection may alter HPV dynamics. A study of Senegalese adult women living with HIV reported a significant reduction in low-risk (not high-risk) clearance of HPV subtypes comparing infections to those uninfected [94]. For adolescents with HIV, a study reported HPV vaccine immunoprotection and set a baseline for future studies on HPV vaccine effectiveness in the context of HIV in youth [95]. Our group has further characterized HPV infection dynamics in CAPHIV [96, 97], showing distinct patterns of oral HPV prevalence, genotype distribution, and immune correlates compared to youth who were perinatally HIV-exposed uninfected and peers who were unexposed/uninfected, findings that underscore the persistent biological impact of early-life HIV exposure on mucosal viral ecology. Studies of adolescents and youth, particularly in countries in SSA, are needed to examine the risk factors and natural history of HPV colonization and persistence, and comprehensively evaluate the benefit of HPV vaccination.

#### Cytomegalovirus and Epstein-Barr Virus (EBV)-associated Infections

In highly endemic areas such as SSA, the interplay between HIV-induced immunosuppression and co-infections with viruses such as cytomegalovirus (CMV) and Epstein-Barr virus (EBV), significantly elevates the risk of malignancies such as Burkitt and Hodgkin lymphomas, nasopharyngeal carcinoma, and other complications [98, 99].

#### Liver Cancers

Perinatal transmission of Hepatitis B virus (HBV) and Hepatitis C virus (HCV) is common in high-endemic areas, and vertical transmission can occur, leading to chronic liver infections from infancy [100, 101]. While ART improves survival in CAPHIV, specific regimens (e.g., protease inhibitors) contribute to liver toxicity, further compounding liver disease risk [102].

### Other Conditions

#### Dermatological Conditions

PLWH experience both infectious and non-infectious dermatoses, though prevalence and severity of dermatological conditions has decreased in the era of improved ART. Mucocutaneous manifestations, pruritic popular eruptions, psoriasis, eczema, bacterial and fungal infections, skin lesions/warts driven by herpes simplex virus, varicella zoster virus, and HPV, are still prevalent with the initiation of ART. ART also has dermatologic side effects, including rashes, lipodystrophy, and pigmentary changes [103, 104]. Limited data are available for CAPHIV. A cross-sectional study in Ethiopia reported skin problems in over 70% of participants [105].

#### Oral/Dental Conditions

CAPHIV continue to face a disproportionately higher prevalence of oral diseases compared to children and adolescents who are uninfected, despite significant successes with ART [106]. Conditions such as oral hairy leukoplakia, linear gingival erythema, Kaposi sarcoma, and candidiasis were rampant during the pre-ART era (prior to 2000) when immunosuppression was often untreated [107, 108]. Although ART has drastically improved overall health outcomes and decreased the prevalence of some of these conditions, emerging evidence from our group and others points to oral health concerns in children and youth affected with HIV, enamel hypoplasia, xerostomia, hyposalivation, salivary gland diseases and dental caries, which significantly impair oral health and quality of life [106, 109–115]. These conditions have been associated with both ART-related shifts in the immune landscape, and the resulting oral microbiome likely compromising the antimicrobial properties of saliva and increase susceptibility to the altered patterns of oral health disorders now observed in CAPHIV [90, 93, 94].

Notably, only a limited number of studies have examined the specific oral health challenges CAPHIV face, making it a critical area for future research [116, 117]. ART created shifts in disease prevalence and oral health patterns underscoring the need for continued focus on comprehensive dental care for PLWH, including CAPHIV on ART, to address the unique challenges posed by both infection and treatment.

## Mechanisms Linking HIV and ART To Chronic Comorbidities in CAPHIV

While many studies have postulated different factors driving the risk of comorbidities described in this review, the underlying mechanistic pathways are not well understood. Chronic comorbidities associated with HIV and ART could be driven by overlapping or distinct biological and immunological mechanisms [118]. Notably, many comorbid conditions that develop during the natural course of aging, including metabolic, neurological, bone, cancerous and dental conditions, are observed at relatively earlier ages in PLWH. The central mechanism driving the increased risk of comorbidities in PLWH is chronic inflammation and immune activation (Fig. 1) [4, 119].

Chronic inflammation is a key component of HIV infection and is a main risk factor for the development and progression of aging and the majority of age-related illnesses [120, 121]. Findings suggest that premature immunosenescence in CAPHIV is mediated by chronic immune activation and inflammation [122], further compounding the burden of comorbidities in this population.

Our review highlights the need for further research and improved therapeutic strategies to mitigate long-term challenges faced by CAPHIV on lifelong ART. Targeted interventions should focus on reducing inflammation, preventing immunosenescence, and preserving overall health, particularly in CAPHIV [119, 123]. Maximizing the potential for healthy aging among children living with HIV on lifelong therapy – particularly in regions most affected – should be a priority [124].

Given that microbial communities are modifiable, profiling microbiota- or aging-mediated processes may offer novel approaches to mitigating HIV-associated immune activation and inflammation [123, 125, 126]. Emerging evidence suggests that even with well-controlled HIV, alterations in microbial communities contribute to a chronic and persistent low-grade inflammation likely driving the progression of age-related conditions in CAPHIV. Moreover CAPHIV receiving ART have been shown to experience epigenetic age acceleration when compared to peers who were uninfected, a phenomenon that has been linked to neurocognitive deficits [7, 127, 128] (Fig. 1). Understanding these interactions may provide new avenues for interventions aimed at promoting healthy aging in CAPHIV.

Below, we briefly highlight two potential biomarkers as molecular tools for monitoring and profiling these lifetime survivors.

### Biological Aging and Epigenetic Age Acceleration

Biological aging in adults with HIV has been documented based on biomarkers of aging including accelerated epigenetic clocks measured by DNA methylation (DNAm) and leukocyte telomere length (LTL) shortening. Telomere shortening has also been reported in CAPHIV in different settings globally, but findings are inconsistent [123, 129, 130].

Epigenetic clocks have emerged as clinically relevant biomarkers of aging which derive estimates of biological age from measures of methylation of cytosine-phosphate-guanine (CpG) sites on DNA, or DNA methylation [5, 131]. Individuals with an increased epigenetic age relative to chronological age have epigenetic age acceleration and have been shown to have increased risk for morbidity and reduced life expectancies. Data on accelerated epigenetic aging is consistent in adults with HIV, but studies focused on CAPHIV are more limited. As this population will experience long cumulative effects of both HIV infection and chronic inflammation, it is important to understand whether increases in epigenetic age acceleration seen in adults are similar in youth with PHIV.

Studies have reported that epigenetic age in CAPHIV under 12 years is accelerated and associated with cognitive impairment [5, 128, 132]. In a cohort of young adults ages 20–35 years with PHIV, negative correlations (*r* = −0.36 to −0.31) between extrinsic epigenetic age acceleration and executive function and attention scores measured by the NIH Toolbox Cognition Battery were observed. Similarly, in older adults with HIV ages 60–82 years, a negative linear relationship between intrinsic epigenetic age acceleration and executive function, attention, and working memory was found [133]. However, less is known about these relationships in adolescence and in African settings. Emerging evidence has demonstrated adolescents with PHIV have accelerated epigenetic aging linked to structural brain changes [127].

### Microbial Profiles in CAPHIV

PLWH undergo significant changes in microbial profiles at various body sites that contribute to chronic immune activation, inflammation, and a higher risk of various chronic conditions. The following section focuses on relevant data on gut and oral microbiome for PLWH and CAPHIV.

#### Gut Microbiome

While total bacterial loads are comparable regardless of serostatus or ART exposure, the gut microbial composition in CAPHIV differ from the uninfected group [134, 135] as also observed in adult PLWH [136–140]. The developing gut microbiomes in infants, children and youth are strongly influenced by maternal factors [141, 142] and by early life factors such as *in utero* HIV exposure [143], suggesting that either HIV or ART, as well as other maternal factors such as antibiotic prophylactic exposure, delivery mode and breastfeeding that could lead to alterations in early-life infant gut microbiome [142, 144, 145]. Early-life colonization of the infant’s mucosal surfaces plays a pivotal role in training and maturation of the immune system [146]. The intestinal microbiome regulates T cell development and subsequently inflammatory immune responses in the infant gut.

The consequences of microbial dysbiosis on immunity and inflammation in HIV infection are linked, and preventing or restoring this altered GI microbiome in HIV infection may have positive impacts on immunity. It is important to understand the origin and consequence of microbial dysregulation in relation to immunological dysfunction [147, 148]. Assessing the association between HIV infection, immune system restoration, and the gut microbiome in children and adolescents with PHIV sheds light on potential avenues for therapeutic interventions.

#### Oral Microbiome

Changes in the oral microbiota have been linked to a variety of unfavorable outcomes, including caries, periodontitis, oral candidiasis, oral herpes lesions, and Kaposi’s sarcoma lesions. These conditions diminish quality of life and can serve as warning indications of an impaired host response and underlying immunosenescence [149, 150]. Maintaining a healthy mucosal barrier, including through host-microbiota interactions, may help alleviate the impact of chronic HIV infection [151].

Although most studies on the oral microbiome have focused on adult PLWH, emerging data from SSA reveal that CAPHIV exhibit distinct salivary microbiome profiles compared to HIV-unexposed/uninfected children [152]. Recent findings also indicate differences in both bacterial and fungal communities in CAPHIV living in SSA [152–154]. Nevertheless, our understanding of how perinatal HIV infection impact the oral microbiota and its association with oral health sequelae in CAPHIV, remains limited.

## Conclusions

CAPHIV appear to be at a higher risk of developing markers of chronic conditions that are typically observed in an aging population compared to their uninfected counterparts. These comorbidities include cardiometabolic conditions, renal toxicity, lung and respiratory dysfunction, growth and developmental disorders, musculoskeletal disorders, mental health disorders, neurocognitive disorders, cancer/malignancies, and other diseases such as dermatologic and oral/dental pathologies. While the profile of comorbidities has changed with improved ART, this increased risk persists and could be due to persistent immune activation, inflammation, and altered immune responses associated with the virus or ART. Several mechanisms likely contribute to the development of these comorbidities in this population, but a deeper understanding of the role that biological aging and microbiome profiles could play in measuring and monitoring these changes across the life course is needed.

Changes in DNA methylation patterns have been linked to immune dysregulation, inflammation, and the development of chronic conditions. Epigenetic changes may contribute to the persistence of inflammation and immune dysfunction seen in these individuals. Similarly, dysregulation of microRNAs in individuals with HIV may contribute to the development of chronic conditions by influencing immune cell function and inflammatory pathways. HIV infection can also lead to alterations in the composition and function of the human microbiome. Microbial dysbiosis particularly in the gut has been associated with chronic immune activation, inflammation, and the development of several age-related conditions described in this review. Disruption of the gut and oral microbiome in CAPHIV can compromise mucosal immunity, leading to increased translocation of microbial products into the bloodstream and systemic inflammation. Further research is needed to use evolving knowledge to develop and shape microbiome-targeted therapies to improve health outcomes in PLWH. The crosstalk between the microbiome and the immune system is essential for maintaining immune homeostasis particularly in early life, childhood and adolescence. Chronic immune activation and inflammation are hallmark features of HIV infection, even in individuals on effective antiretroviral therapy. Persistent immune activation can lead to tissue damage, organ dysfunction, and the development of comorbidities such as cardiovascular disease, neurocognitive disorders, and metabolic conditions. Dysregulated inflammatory pathways, including the production of pro-inflammatory cytokines and activation of immune cells, contribute to the pathogenesis of chronic conditions in children and adolescents living with HIV. Targeting these inflammatory pathways may offer therapeutic opportunities for managing and preventing comorbidities in this population.

Understanding the interplay between accelerated epigenetic aging, the microbiome, immune activation, and inflammation is crucial for elucidating the mechanisms driving chronic conditions in children and adolescents living with HIV. Future research exploring these intricate relationships may uncover new therapeutic targets and interventions aimed at mitigating the burden of chronic comorbidities in individuals with HIV.

## Supplementary Information

Below is the link to the electronic supplementary material.

Supplementary Material 1

## Data Availability

No datasets were generated or analysed during the current study.
